# Parliament and the Professions

**Published:** 1920-08-21

**Authors:** 


### Parliament and the Professions.

### Third London General Hospital.

In answer to a question, Dr. Addison said that he had ascertained that the site of the Third London General Hospital is held by the London County Council on trust for the purpose of an open space; that it was acquired by them before the war for that purpose from the Royal Patriotic Fund Corporation at less than market value in consideration of the use to which it was to be put; and that the Royal Patriotic Fund Corporation object to its use for a civilian hospital, and urge that the conditions on which the land was sold must be adhered to. Part of the price paid for the land was subscribed locally, and there is strong opposition to the land being diverted from use by the public as an open space. Legislation would be necessary in order to secure the retention of the hospital on its present site, and as in the circumstances Dr. Addison did not think that Parliament would be willing to give the necessary powers, he had regretfully come to the conclusion that he must abandon his effort to obtain the use of the hospital on its present site for the civilian population.

